# Supplementary material for: Graphene Oxide Dielectric Permittivity at GHz and Its Applications for Wireless Humidity Sensing
Source: Sci Rep. 2018 Jan 8;8:43. doi: 10.1038/s41598-017-16886-1 (PMC5758609; doi:10.1038/s41598-017-16886-1)
Supplement: Supplementary file 1 — Supplementary Information [file 41598_2017_16886_MOESM1_ESM.pdf]

Supplementary Information

Graphene Oxide Dielectric Permittivity at GHz and Its Application for Wireless Humidity Sensing

[1]Xianjun Huang, [1]Ting Leng, [2]Thanasis Georgiou, [3]Jijo Abraham, [3,4]Rahul Raveendran Nair, [3,4]Kostya S. Novoselov, [1,4]Zhirun Hu*

*Correspondence: Zhirun Hu (z.hu@manchester.ac.uk)

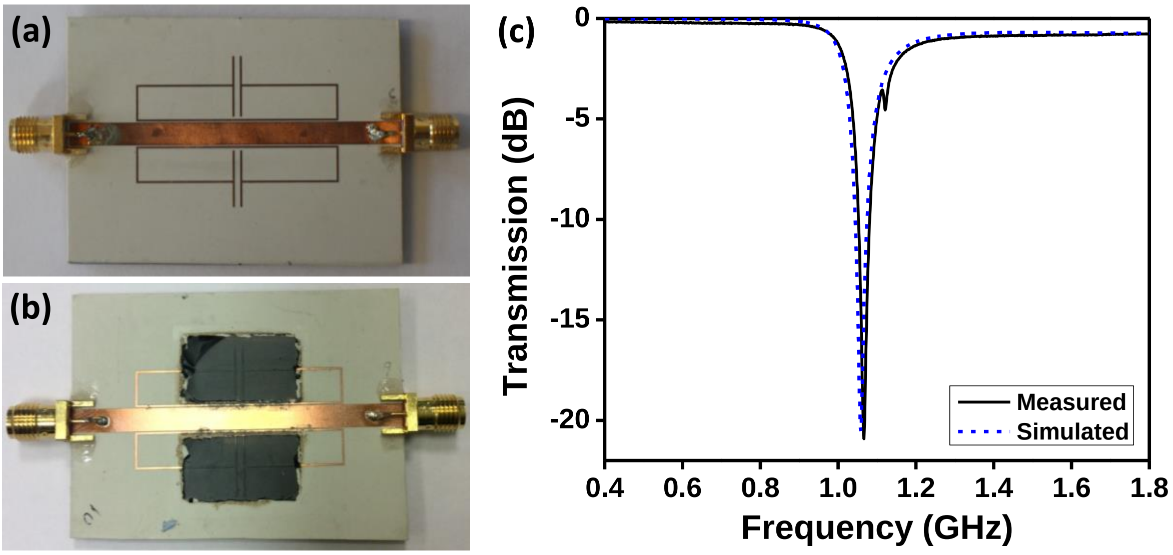

Fig. S1. (a) Microstrip resonator without printed GO layer, (b) Microstrip resonator with printed GO layer ($15\,mm \times 8\,mm$). The thickness the GO is 30 μm and (c) Simulated and measured transmission coefficients (S_{21}).

Fig. S1 shows the designed resonator for GO permittivity measurement and extraction. To validate the full electromagnetic wave simulation (CST Microwave Studio), we have compared the simulated and measured transmission coefficients of the resonator without GO layer, Fig. S1 (c). It can be seen that the simulated results agree very well with the measured ones, validating the simulation.

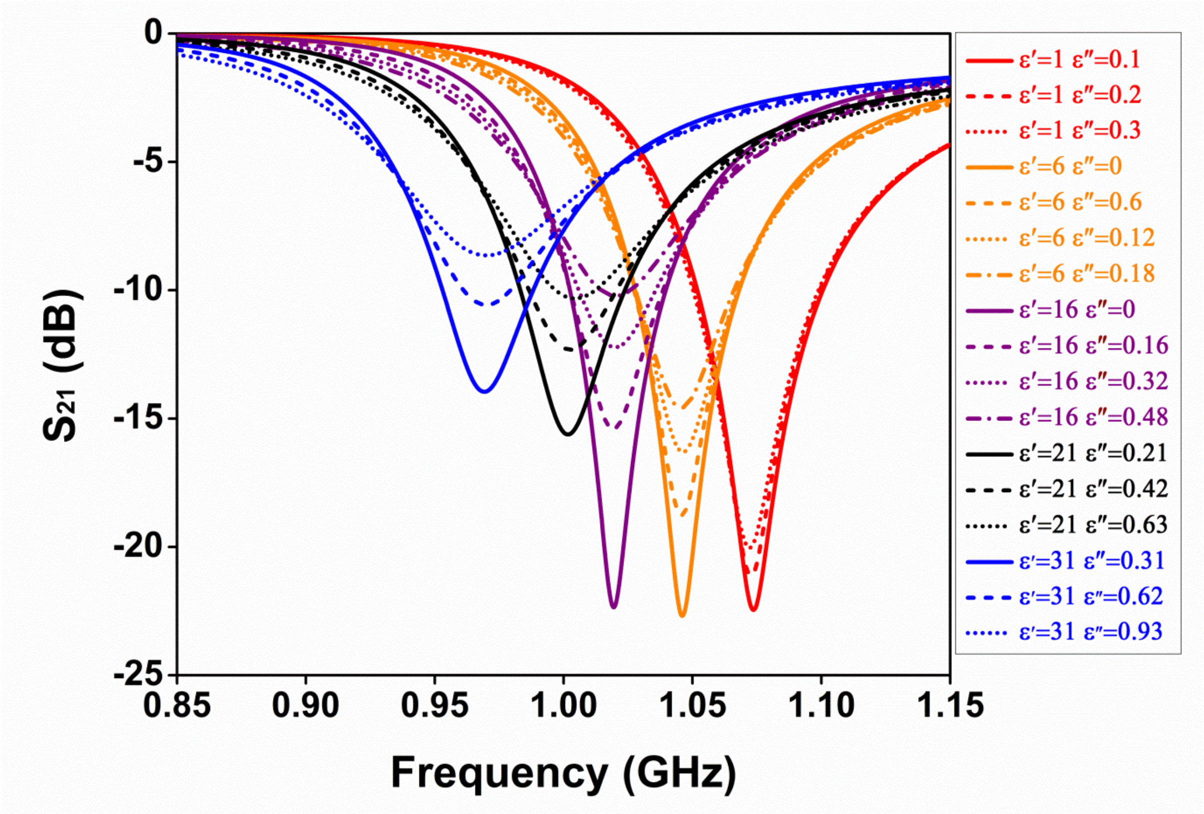

Fig. S2. Simulated transmission coefficients (S_{21}) of the resonator with dielectric layer of various relative permittivity (ε' and ε'').

To extract the relative permittivity of the GO under various humidity conditions, GO was mimicked by thin dielectric layer, which has exactly the same size, thickness and location as that shown in Fig. S1 (b). The transmission coefficients of the resonator of 5 different sets of relative permittivity ($\varepsilon_r = \varepsilon' - \mathrm{i}\varepsilon''$) were simulated and shown in Fig. S2. Each colour set of the curves in the figure contains the same real part (ε') but various imaginary part (ε'') of the relative permittivity. It can be observed that for the same ε', the resonance frequency changes little with ε''. This is because ε'', which is related to material loss tangent ($tan\delta = \varepsilon''/\varepsilon'$), mainly affects the Q factor of the resonator. The simulations reveal that the changes of relative permittivity pose obvious alteration on the resonator's transmission performance. GO permittivity can be extracted by comparing the experimental measurements and full electromagnetic wave simulations.

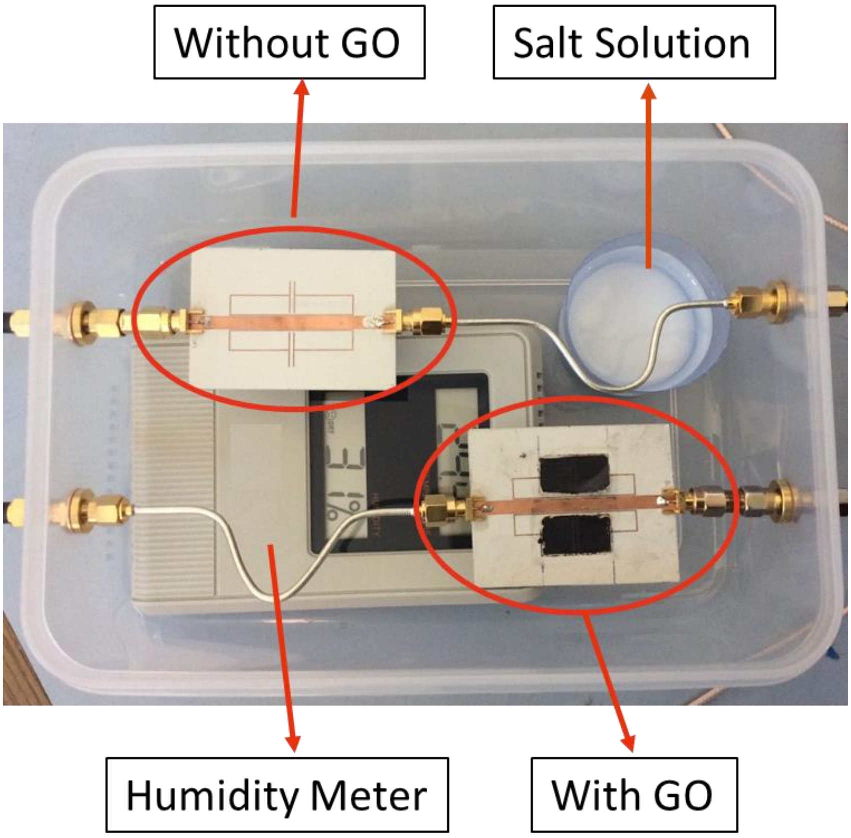

Fig. S3. Sealed box setup for GO permittivity measurement (the top cover of the box has been removed for a better view).

Fig. S3 shows the GO permittivity measurement setup. A digital humidity meter (Fisher Scientific 116617D) is placed in the box for monitoring. Rubber-tight SMA connectors (RS Stock No.716-4798) are used to connect inside/outside microwave cables, making the box well sealed. The out-extended cables are connected to VNA (Agilent E5071B) for scattering parameter (S-parameter) measurements.

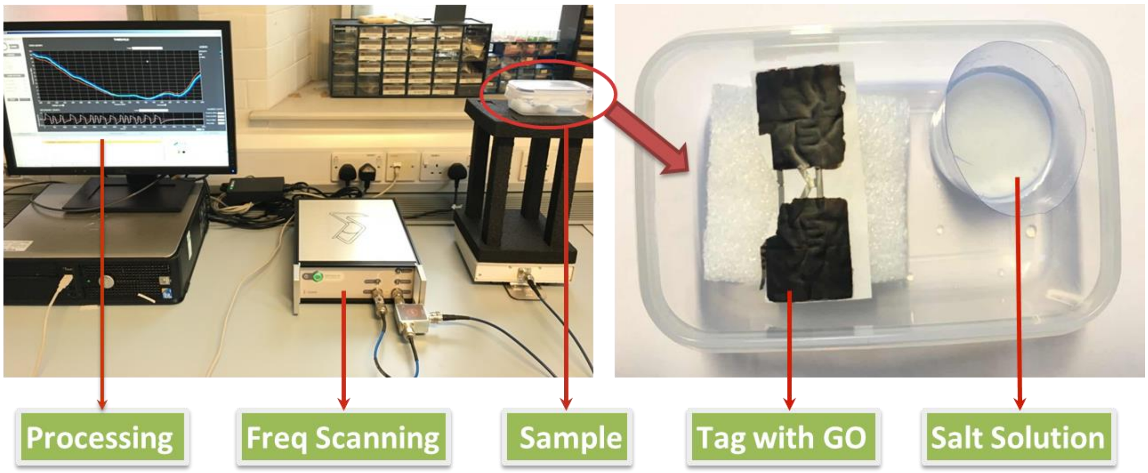

Fig. S4. Experimental setup for wireless RFID GO humidity sensing system.

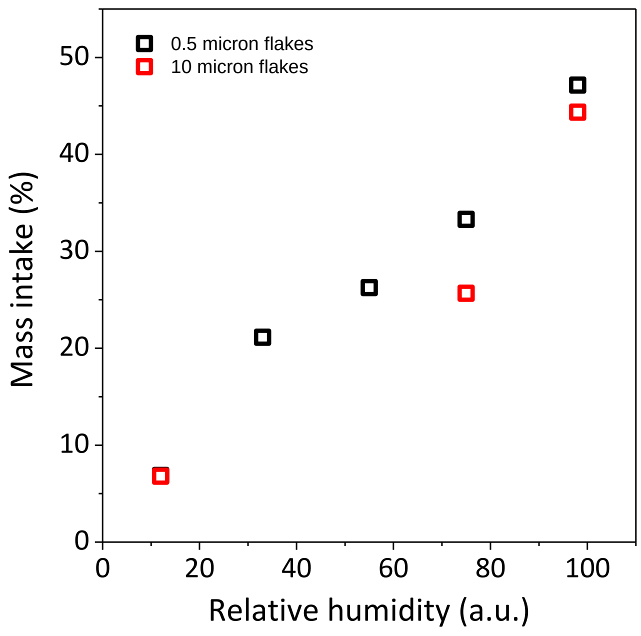

Fig. S5. Water uptake in GO samples. Relative increase in weight of GO prepared from different flake sizes exposed to different humidity.

Fig. S5 shows the measurement results of water uptake of GO with two different flake sizes (0.5 μm and 10 μm) by monitoring the weight change of GO exposed to different humidity conditions.

GO samples were completely dried inside a glove box before exposing to different humidity for 5 days. Mass uptake monotonically increases with increasing humidity and it varies from ~ 5% to 50%.

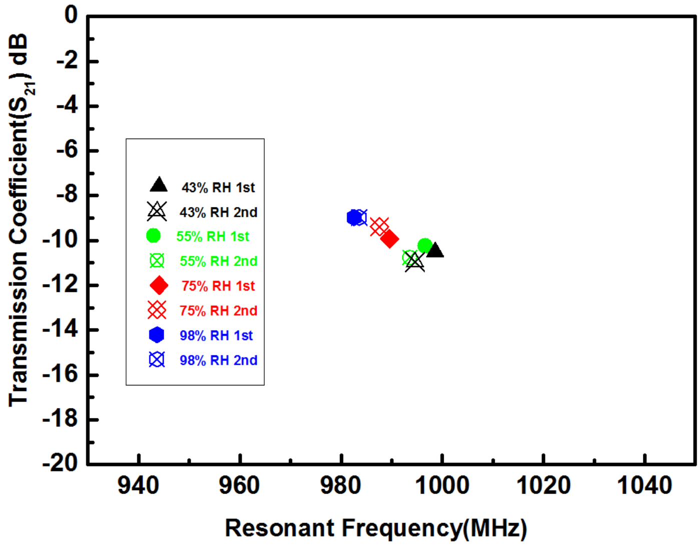

Fig. S6. Durability test of frequency response (x axis) and transmission response (y axis) of the GO coated resonator for various RH.

Fig. S6 shows the durability test of the resonator coated with GO for 43% RH to 98% RH. The first measurement was taken before drafting the manuscript in December 2015. The second measurement was made in January 2017. The time span is 13 months. All the measurement data collected were after 96 hours of humidity equilibration time. The x axis is the resonance frequency which indicates the frequency response to the GO coated resonator. The y axis is the transmission coefficient which indicates the propagation of the GO coated resonator. It can be seen that the measured results agree well with the previous data in both frequency response and the propagation level.

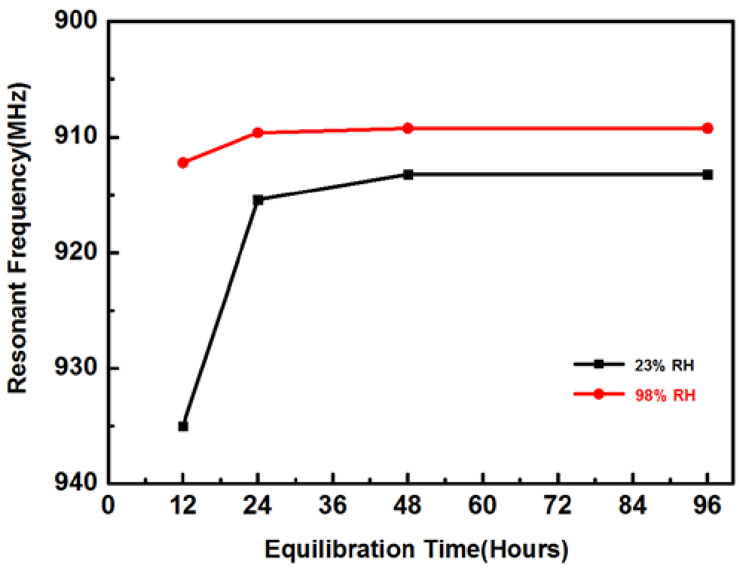

Fig. S7. Stability test of resonance frequency of GO coated sensor over equilibration time in room temperature (RH=23%) and with K_2SO4, (RH=98%).

Fig. S7 shows the measured stability of the GO sensor. The resonance frequencies of GO coated sensor were measured with 23% RH and 98% RH, respectively. After 12 hours of equilibrium time, the resonance frequency still changes. The resonance frequency becomes stable and shifts no more after 48 hours.
